# Annotations

**Published:** 1909-11-13

**Authors:** 


### ANNOTATIONS.

### The Responsibility of Silence.

This subject was dealt with in The Hospital of the 16th ult. It often happens that points arise where a chairman or a member of committee learns of some matter or proceeding which he may consider strange, or even improper. In such cases there is no doubt that his plain duty is to bring this at once to the notice of the Committee of Management, and for that committee, dealing with it with closed doors, to take steps to refer it, when necessary, to the Medical Board—and every hospital should have a properly constituted Medical Board—for report and opinion. A matter which occurred at Inverness, as reported in the *Dundee Courier* of the 4th instant, seems to be a case in point. A special committee of the directors investigated a charge brought against a member of the medical staff of neglect of a patient. The case has excited a great deal of interest in Northern England, and the report of this committee forms a lengthy and important document. It makes it clear that the relations between certain members of the staff are strained, and that there is a want of harmony which ought not to be permitted to continue in a hospital. For some unexplained reason the report appears to have been rendered abortive by the action of the Provost, who presided at the meeting. He refused to permit any motion to be proposed, and treated the submission of the report as a mere act of courtesy to the managers. We venture to think that this is a striking instance of the Responsibility of Silence which will not contribute to the well-being of the Northern Infirmary, or of anybody connected with it. The admission of healthy public opinion, and its expression in concrete form, ought not to be rendered nugatory in this way. We hope that the managers, or some of them, will call a special meeting and record the opinion of the majority, so as to secure that any defects in the management or neglect in the treatment of patients at the Northern Infirmary may be rendered impossible for the future.

### The Birthday Honours.

The Birthday Honours list, so far as the medical profession is concerned, is scarcely a very interesting one. Among the new baronets is Mr. James Roberts, whose charitable gifts to Yorkshire hospitals have made his name known in the institutional world. Among the new knights is Dr. Pollard, who is a barrister of the Inner Temple, and represents Eccles division in the House of Commons. He graduated M.D. with honours at Edinburgh in 1890, and was for a time assistant to the Professor of Midwifery, R.C.S., Edinburgh. He has served as Mayor of Southport, where he practises, and as member of the Lancashire County Council. Mr. Francis Belsey, another new knight, is well known for his charitable work, while Mr. James Moody, who also gets a knighthood, is the well-known superintendent of the L.C.C. Cave Hill Asylum in Surrey. Dr. James Haran, medical officer of health at Mombasa, East African Protectorate, who gets a C.M.G., graduated at Dublin in 1891. Sir Savile Crossley, Bart., the well-known Honorary Secretary of King Edward's Hospital Fund and Chairman of the Hospital Saturday Fund, is promoted to a Knight of the Royal Victorian Order, of which he has been a member. Not the least interesting announcement in the list is the brief mention that the King has been graciously pleased to confer the decoration of the Royal Red Cross upon Miss Annie Fletcher, who has been a hospital nurse for twenty years, in recognition of devoted service rendered by her to His Majesty and Her Majesty the Queen since 1902.

### Quacks and Gulls.

Despite the verdict, and despite clumsy attempts at an Autolycus-like humour on the part of the defendant, there is a good deal in the reports of a recent action to sadden the thoughtful medical man. At a time when hospitals are particularly short of money, and medical research is rubbing along as best it can, here is fresh evidence, if it were needed to convince, how mischievously impostors waste resources which should be sacred to the relief of suffering and the advance of knowledge. It is not so much, however, on the revelations concerning Mr. Bodie's way of livelihood that we rely to point a moral, as on the tone of the Judge's summing up. Until instruction in elementary anatomy and physiology becomes *de rigueur* in every decent public school, probably there will continue to be heard such assertions from the Bench as that "a person who had not gone through the hospitals can develop a certain amount of skill in breaking down adhesions." Well, such a person can, as the following case, which we happen to know of, shows. A working man with a bent, ankylosed knee, the result of tubercle (practically not much more of a disability than the club foot of Hippolyte in *Madame Bovary*), had it forcibly straightened by a bonesetter. The old disease lighted up, and amputation became necessary. Members of the legal profession should remember that the knowledge of the world of which they are so proud must necessarily own some limitations.

### Our Commission on Beer and Stout.

It would appear from certain correspondence and numerous paragraphs in the lay press that the Report of our Special Commission on Beer and Stout has stirred up some political bias. We very much regret this, because we have nothing to do with politics. Our Report on Beer and Stout is a scientific document based on facts. We are perfectly willing and ready to deal with any criticism of this Report appearing in a scientific or medical paper. It is, however, out of the question for The Hospital to take notice of comments appearing in the lay press. One correspondent who has written us a number of letters makes what he describes as a "challenge," involving a deposit of money on both sides. The idea of accepting such a "challenge" is ridiculous. The point which appears to have annoyed these critics is the comparison between stout and milk and bread, but if they will obtain Dr. Robert Hutchison's recognised Text-book on Dietetics they will find that it makes the same comparison and in the same sense.

